# The myokinetic control interface: tracking implanted magnets as a means for prosthetic control

**DOI:** 10.1038/s41598-017-17464-1

**Published:** 2017-12-07

**Authors:** S. Tarantino, F. Clemente, D. Barone, M. Controzzi, C. Cipriani

**Affiliations:** Scuola Superiore Sant’Anna, The BioRobotics Institute, V.le R. Piaggio 34, 56025 Pontedera (PI), Italy

## Abstract

Upper limb amputation deprives individuals of their innate ability to manipulate objects. Such disability can be restored with a robotic prosthesis linked to the brain by a human-machine interface (HMI) capable of decoding voluntary intentions, and sending motor commands to the prosthesis. Clinical or research HMIs rely on the interpretation of electrophysiological signals recorded from the muscles. However, the quest for an HMI that allows for arbitrary and physiologically appropriate control of dexterous prostheses, is far from being completed. Here we propose a new HMI that aims to track the muscles contractions with implanted permanent magnets, by means of magnetic field sensors. We called this a myokinetic control interface. We present the concept, the features and a demonstration of a prototype which exploits six 3-axis sensors to localize four magnets implanted in a forearm mockup, for the control of a dexterous hand prosthesis. The system proved highly linear (R^2^ = 0.99) and precise (1% repeatability), yet exhibiting short computation delay (45 ms) and limited cross talk errors (10% the mean stroke of the magnets). Our results open up promising possibilities for amputees, demonstrating the viability of the myokinetic approach in implementing direct and simultaneous control over multiple digits of an artificial hand.

## Introduction

Restoring dexterous motor functions equivalent to those of the human hand, after amputation, is perhaps one of the major goals in rehabilitation engineering and applied neuroscience. Reaching this goal requires the successful achievement of the two pivotal components of a prosthesis: the artificial hand and the Human-Machine Interface (HMI). While the artificial hand should be capable of movements and grasps comparable to those of the natural hand, the HMI should allow intuitive/effortless control of such movements, forming a bridge between the artificial device and the sources of volition. The HMI is the primary focus of this work.

Intuitive prosthetic control may be achieved by extracting the amputee’s intention from signals recorded in non-invasive or invasive ways from the Peripheral Nervous System (PNS). Many attempts to tap into these neuromuscular signals have been made, ranging in invasiveness (from surface sensors to intra-neural electrodes) and hierarchical location (skeletal muscles, PNS) (see Micera *et al*. for a review^1^). Nonetheless still today the most reliable and clinically viable technique is the use of the electromyogram (EMG), picked up by surface electrodes to control the movements of an electromechanical prosthesis. In fact, the envelope of the EMG signal is broadly proportional to the level of contraction of the muscle being recorded^2^. Commercially available *myoelectric* prosthetic hands are typically controlled by a single agonist/antagonist EMG pair that controls hand opening and closing (all digits together). This kind of control is called direct control and is the most primitive, simple, fast and robust approach. In direct control the user voluntarily contracts one muscle in the absence (independently) of all others: i.e. each EMG signal is mapped to a unique function. Potentially, if multiple independent muscles are accessible, direct control can entail simultaneous control of multiple DoFs within a prosthesis, in a physiologically appropriate manner^3,4^. However, this possibility remains an intriguing solution shown in research laboratories and acute (non-chronicle) settings. As currently implemented, surface EMG (sEMG) does not provide simultaneous control of multiple DoFs, due to the lack of accessible independent control sources. In turn, this type of control can be very slow, unintuitive and inefficient^5^.

To overcome this issue, *pattern recognition* was widely explored since the sixties as an alternative approach to direct control^6,7^. It is based on the premise that amputees can voluntarily activate distinct contractions in their residual muscles, and that algorithms can map the associated signal patterns to classes of motor commands to the prosthesis. Although pattern recognition was proven effective in research and clinical settings^7,8^, the approach is neither physiologically appropriate nor dexterous because arbitrary movements or hand postures cannot be obtained^7,9^.

Novel techniques aiming to improve the number of accessible and independent signals for control have been, and are being, developed and assessed in the last decade^10^. Such new approaches span from implantable interfaces that record the signals closer to their biological sources (like the implantable myoelectric sensors – IMES^11^ or epimysial electrodes^12^), to surgical techniques that create additional sites for sEMG (i.e. targeted muscle reinnervation – TMR^13^) or that regain access to the natural neuromuscular structures (e.g. the osseointegrated human-machine gateway^12,14^). In several instances these approaches are combined.

The IMES, developed by Weir and colleagues, is perhaps among the most promising interfacing solutions^11^. IMESs are small implantable electrodes intended to record and wirelessly transmit intramuscular EMG to a hand prosthesis. Multiple IMESs may capture simultaneous signals from multiple muscles in the residual limb with minimum cross-talk, allowing natural and direct control of multiple DoFs in parallel. IMES-based prostheses hold the promise to solve many of the clinical issues affecting sEMG, and are currently undergoing clinical trials. In particular, the first demonstration of a system exploiting six IMESs in a transradial amputee was recently presented^15^. Nonetheless, IMESs are active electronic devices which require a power supply through a wireless inductive coupling having a low power transfer efficiency^16,17^. In fact, the system that is currently in human clinical trials uses 70% of the prosthesis battery capacity to generate the electromagnetic field for wireless power coupling to the implanted sensors^15,17^.

**Figure 1:**
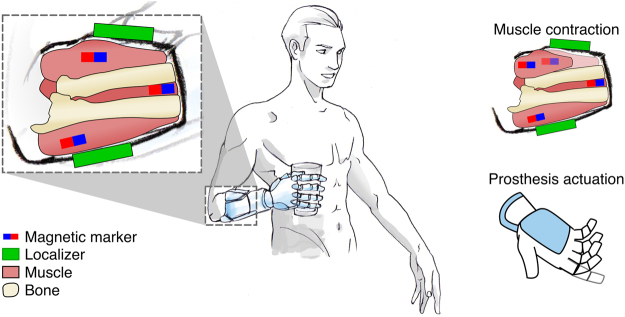
Overview of a myokinetic control interface for a prosthetic hand. Magnetic markers are implanted in relevant wrist muscles and extrinsic muscles of the hand; the voluntary contractions of these muscles are monitored by the localizer which estimates the position of the magnetic markers. This information is used to control the physiologically appropriate degree of freedom of the prosthetic wrist and hand in a direct and proportional manner.

Here we propose a new approach that by abandoning the paradigm of transducing electrical signals, embraces the idea of sensing the magnetic field of implanted magnetic markers (MMs), to control multiple DoFs in a prosthesis, akin to IMESs. By implanting into a muscle a passive MM, namely, a permanent magnet, it could be possible to localize its position using external magnetic sensors. In particular, it could be possible to detect the muscle deformation during contraction (some contract up to 30–40% of their length)^18^. Indeed, since the MM would travel with the muscle it is implanted in, its localization would provide a direct measure of the contraction/elongation of that muscle (which is voluntarily controlled by the central nervous system). During isotonic contractions, the main deformation of the muscles is observed in their axial direction (they shrink). During isometric contractions (i.e. when the muscle generates force because the distal end is blocked), the muscle mainly deforms in the radial direction (i.e. it bulges). By monitoring both these (radial and axial) deformations, in principle it is possible to retrieve information on the force exerted by the muscle and on the joint position^2,19^, and this information could be used to control the force/position of the relative DoF in the hand prosthesis. Generalizing, multiple MMs in multiple muscles, could entail **simultaneous**, **physiologically** **appropriate**, **direct control** over **multiple** DoFs. If successful, this approach could have an impact on and beyond transradial amputees because it can be extended to all kinds of upper limb and lower limb amputations, and combined to surgical procedures like TMR for a more stable interface. Notably, this could be possible using passive implants not requiring a power supply and not subject to electrical failures and maintenance. As the envisioned HMI would transduce residual muscle movements into decipherable signals, we borrowed from the Greek roots and called it *myokinetic* control interface (Fig. 1).

In this work we present the concept of the myokinetic interface, together with its design features, and a proof of concept of a prototype which exploits six 3-axis magnetic field sensors to localize four MMs in an anatomically relevant workspace. In fact, the MMs were mounted onto artificial muscles in a forearm mockup, designed to replicate the anatomical placement and contraction movements of the extrinsic muscles of the hand. In particular, the MMs were mounted on the emulated Flexor Pollicis Longus (FPL), on the first two compartments of the Flexor Digitorum Superficialis (FDS1 and FDS2) and on the Abductor Pollicis Longus (APL) so as to control the flexion of thumb, index, and middle fingers and the abduction of the thumb in a multi-DoF prosthetic hand, respectively. The mockup was used to experimentally assess the accuracy, repeatability and response time of the myokinetic interface prototype. Finite Element (FE) modelling used to assess the effects of external magnetic interferences and their attenuation using shields, complemented the analysis. Finally, the potential of the myokinetic interface as a control system of simultaneous and independent DoFs of a prosthesis was assessed online using a data-glove and a robotic hand.

## Architecture of a myokinetic control interface

A myokinetic control interface comprises (i) multiple MMs, each one implanted in one target muscle, and (ii) a localizer hosted in the prosthetic socket ideally constrained to the skin (Fig. 1). The localizer is responsible for continuously tracking the position and the orientation of the implanted MMs, using magnetic field sensors and solving the so called magnetic inverse problem. In the case of a single MM, the theoretical magnetic field ***B***_*i*_ at the position of the i^th^ sensor, is modelled after the vectorial equation of the magnetic dipole^20^:

1 $${{\bf{B}}}_{i}=\frac{M{\mu }_{r}{\mu }_{0}}{4\pi }(\frac{3(\hat{{\bf{m}}}\cdot {x}_{i}){x}_{i}}{{|{x}_{i}|}^{5}}-\frac{\hat{{\bf{m}}}}{{|{x}_{i}|}^{3}})$$

where *M* and ***m*** are the magnitude and direction of the magnetic moment of the MM, respectively; ***x***_***i***_ is the vector distance between the MM and the i^th^ sensor.

In the case of *N* sensors and *n* magnets, ***B***_*i*_ at the location of the i^th^ sensor can be modelled as the linear superimposition of that generated by each dipole. Thus, for each i^th^ sensor, the following equation applies:

2 $${{\bf{B}}}_{i}=\sum _{j=1}^{n}\frac{{M}_{j}{\mu }_{r}{\mu }_{0}}{4\pi }(\frac{3({\hat{{\bf{m}}}}_{{\bf{j}}}\cdot {x}_{ij}){x}_{ij}}{{|{x}_{ij}|}^{5}}-\frac{{\hat{{\bf{m}}}}_{{\bf{j}}}}{{|{x}_{ij}|}^{3}})$$

where j indicates the j^th^ magnet. Reversing Equations 1 or 2 provides the absolute position of one or *n* MMs (notably the reverse problem has no closed form, thus numerical approximation is required). In fact, the absolute position of a MM is not informative alone of the status of the contraction of a residual muscle. It is the displacement of the MM from an offset position (recorded with uncontracted, relaxed muscles) that reveals the degree of contraction of the muscle it is implanted in. Hence the localizer is responsible for estimating *displacements* relative to a rest state.

In principle, the estimated displacement provides an information of the force applied by the muscle itself^2,19^ and could be used to control the force/position of the associated DoF in a powered prosthesis (i.e., direct control). However, the estimation may be affected by modelling and numerical approximations, technological limitations, and environmental factors, which may degrade both the accuracy and the precision (repeatability) of the control signals.

Modelling and numerical approximations are introduced by the magnetic dipole model, which treats the MMs as ideal and pointlike dipoles, and by computational errors introduced when reversing the model. These cause errors in localizing single magnets (E_model_) and can yield to false predictions of simultaneous displacements (i.e. cross-talk between control signals – E_cross-talk_) in the case of multiple magnets. Technological limitations refer to the accuracy and repeatability of the magnetic field sensors (E_sensor_). Environmental factors include the geomagnetic field (E_geo_), external magnetic fields produced by localized magnetic sources (E_local_), and the effects of relative compression/depression movements or misalignments between the socket (where the localizer is) and the stump tissues (where the implanted MMs are – E_shift_). Considering all these factors, for a myokinetic control interface comprising *n* magnets, the displacement of a *MM*_*x*_ as estimated by the localizer (D_m_) can be expressed as:

3 $${D}_{m}^{M{M}_{x}}={D}_{a}^{M{M}_{x}}+{E}_{{\rm{model}}}+\sum _{y=1}^{n-1}{E}_{\mathrm{cross} \mbox{-} \mathrm{talk}}^{y}+{E}_{sensor}+{E}_{geo}+{E}_{local}+{E}_{shift}$$

where *D*_*a*_^*MMx*^ is the actual displacement of *MM*_*x*_. Notably the geomagnetic field is a common mode signal and thus E_geo_ can be readily cancelled out using differential measurements with respect to a remote sensor not subject to the magnetic field produced by the MMs. In the present work, we have quantified the effects of the above mentioned approximations, limitations and factors on the performance of the myokinetic control interface, by assessing E_model_, E_cross-talk_, E_sensor_ and E_local_, while using differential measurements as to cancel out E_geo_.

## Results

### Effects of modelling approximations and technological limitations

**Figure 2:**
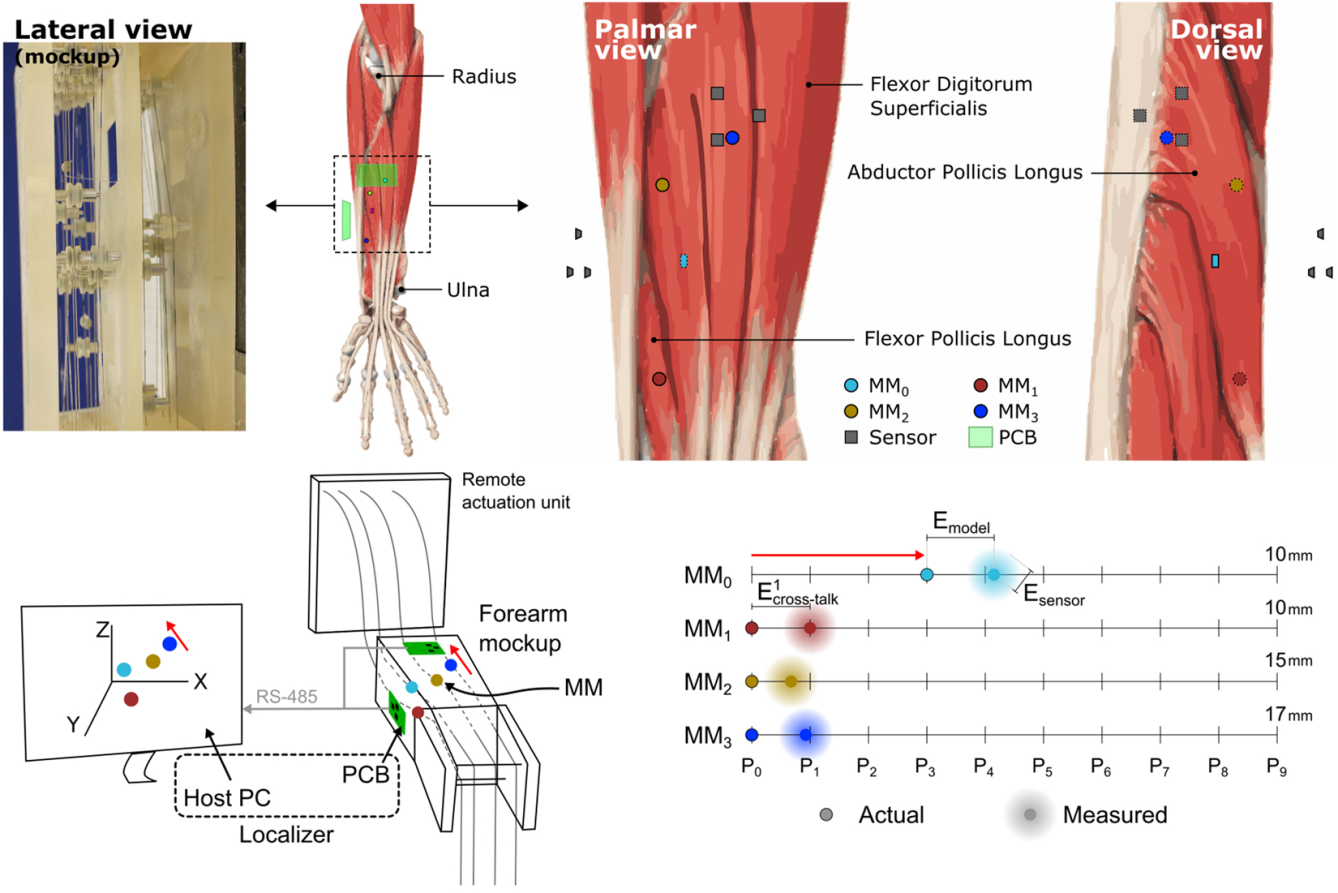
The experimental setup. Upper panel: The forearm mockup with *implanted* magnets (MMs). The mockup aimed at reproducing the natural position and orientation of forearm muscles, in addition to their deformation due to contraction. The mockup replicated the movements of 17 degrees of freedom of the hand and wrist^33^, for a total of 17 wires, albeit only four were used. Muscles were modelled using a wire attached on one side to a servo motor (housed in a remote actuation unit) used to actively *contract* the muscle. Four cylindrical MMs were *implanted* on the abductor pollicis longus (MM_0_), flexor pollicis longus (MM_1_) and compartments 1 and 2 of the flexor digitorum superficialis actuating the index (MM_2_) and middle fingers (MM_3_). Lower panel: The localizer, composed of two custom PCBs and a host PC, was used to track the position of the MMs solving the magnetic inverse problem. E_model_ indicates the errors due to the magnetic dipole model. E_cross-talk_ indicates the error due to cross-talk between MMs. E_sensor_ indicates the error introduced by the six magnetic field sensors (i.e. electronic noise). P_0_ indicates the rest position of the MMs (i.e. relaxed muscle); P_9_ corresponds to maximum muscular contraction and depends on the muscle where the MM is implanted in. In order to assess E_model_, E_cross-talk_, E_sensor_, affecting the myokinetic interface, each MM, one at a time, was moved along the entire range of motion (ROM) of the emulated muscle, and data was collected at the ten equidistant points in space P_0_, …, P_9_.

A localizer based on six 3-axis magnetic field sensors implementing the Levenberg-Marquardt optimization method was used to solve the magnetic inverse problem and estimate the displacements of the four *implanted* MMs^21^ (Table 1 and Fig. 2). In order to assess E_model_, E_cross-talk_, E_sensor_, affecting the myokinetic interface, each MM, one at a time, was moved along the entire range of motion (ROM) of the emulated muscle, and data was collected at ten equidistant points in space (P_0_, …, P_9_ corresponding to relaxed muscle, …, maximum contraction) along the muscle trajectory (Fig. 2, lower panel).

**Table 1:** Inter-MM and MMs - closest sensor distances.

Emulated muscle	Distance of	from MM_1_	from MM_2_	from MM_3_	from closest sensor
Abductor Pollicis Longus	MM_0_	33.4	40.9	53.3	29.2
Flexor Pollicis Longus	MM_1_	—	52	70.9	26.3
Flexor Digitorum Superficialis (compartment 1)	MM_2_	—	—	24.6	31
Flexor Digitorum Superficialis (compartment 2)	MM_3_	—	—	—	28.7

All measures in mm.

For each MM, E_model_ was computed as the mean Euclidean distance between the actual and the estimated displacement (Fig. 2). E_sensor_ was computed as the mean modulus of the standard deviation vector of the computed displacement. E_cross-talk_ was calculated as the mean displacement (across the ten positions) computed by the localizer (Fig. 2), for each of the three MMs kept at P_0_.

**Figure 3:**
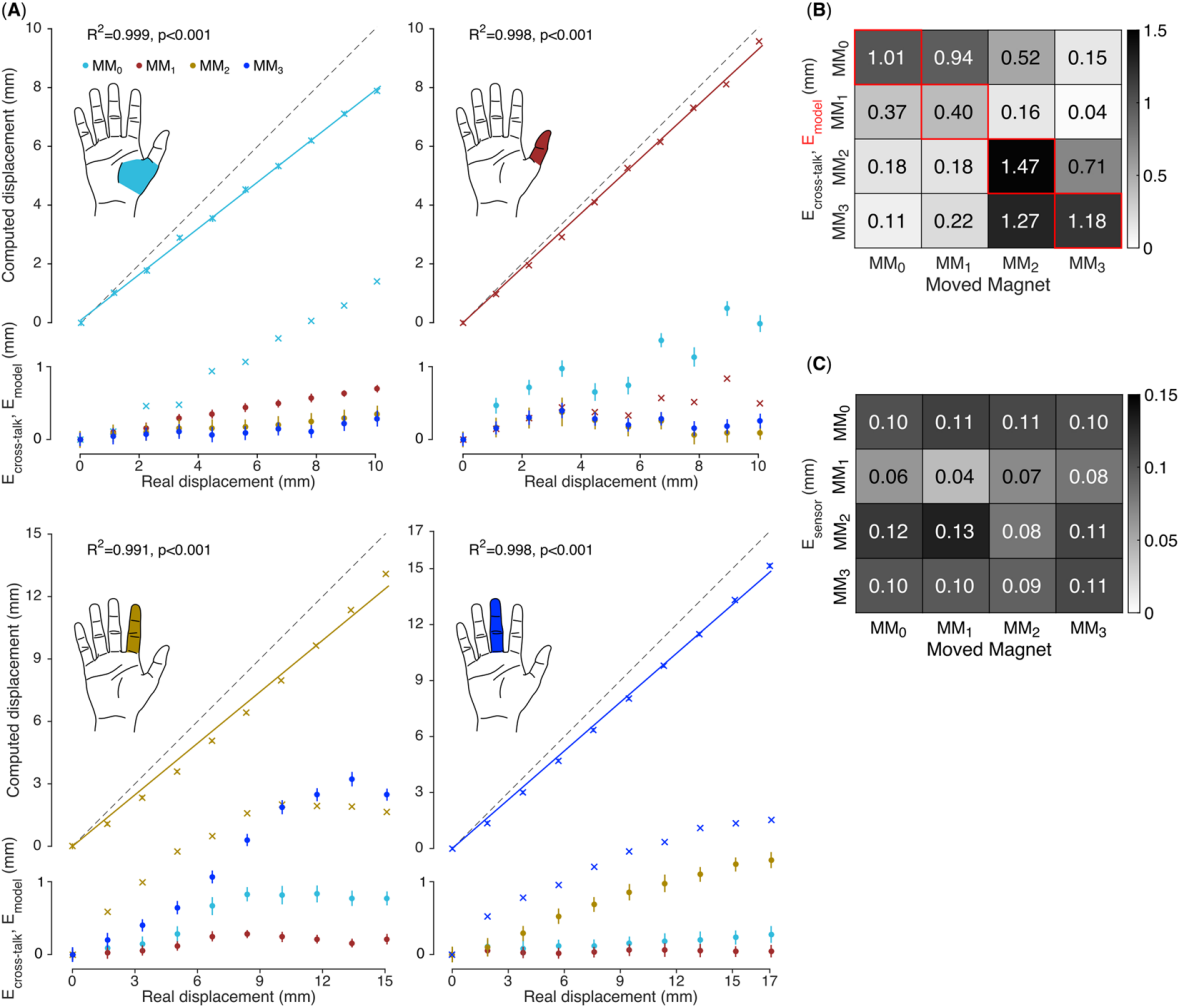
Performance of the myokinetic experimental prototype: E_model_, E_cross-talk_, E_sensor_. Each MM was moved along the anatomically appropriate ROM of the relative muscle (a: MM_0_ – APL; b: MM_1_ – FPL; c: MM_2_ – FDS_1_; d: MM_3_ – FDS_2_), while the remaining MMs were kept at their initial/rest position. (**A**) Estimated versus actual displacements of the MMs: while the moving MM is appropriately tracked (R^2^ = 0.99) the computed displacement of non-moving MMs represent the cross-talk errors, E_cross-talk_ (dot markers). For each moving MM,the difference between the actual and the estimated displacement (E_model_) is shown with cross markers. (**B**) E_model_ and E_cross-talk_ averaged over the entire ROM. E_model_ and E_cross-talk_ correspond to the diagonal and non-diagonal elements of the confusion matrix, respectively. (**C**) Technological limitations affecting the myokinetic interface (E_sensor_). For both panels (**B**) and (**C**): each row contains the errors related to a single MM; each column contains the errors generated by the movement of a specific MM.

For all MMs, E_model_ was found to range between 0.15 mm and 2.1 mm (Fig. 3A). Additionally, it increased with distance from the rest position P_0_ for all MMs. The largest E_model_ was found to be 2.2 mm, for the *abductor pollicis longus*, corresponding to 22% of its anatomical ROM. The relationship between the actual and the computed displacement was found to be highly linear for all MMs (R^2^ = 0.99, p < 0.001, Fig. 3A).

The movement of one MM slightly affected the position estimation of the other MMs. The importance of this effect varied consistently depending on which MM was moved and its distance from the other MMs. Indeed, the maximum E_cross-talk_ (2.4 mm, corresponding to 16% the ROM of the FDS_1_, Fig. 3A) was measured on the estimate of MM_3_ while moving MM_2_, which were the magnets being closer to each other (Table 1). Distances between MMs above 40 mm (Table 1) exhibited minimal E_cross-talk_ (i.e. < 0.5 mm, Fig. 3A, equivalent to ~3% of the muscles ROM).

The mean E_model_ (averaged across the entire ROM) always proved lower than 1.5 mm (Fig. 3B, diagonal elements). This corresponds to 14.7%, 14.7%, 9.8% and 8.6% of the entire ROM of MM_0_, MM_1_, MM_2_ and MM_3_, respectively. The mean E_cross-talk_ (Fig. 3B, non-diagonal elements) proved lower than 1.27 mm, i.e. 12.7%, 12.7%, 8.4% and 7.4% of the entire stroke of MM_0_, MM_1_, MM_2_ and MM_3_, respectively.

E_sensor_ was found to be independent from the spatial distribution of the MMs (Fig. 3C). Indeed, it was almost the same for each MM regardless if other magnets were moving or not. The mean E_sensor_ was 105.3 µm, 62.8 µm, 109.4 µm and 98.6 µm for MM_0_, MM_1_, MM_2_ and MM_3_, respectively (Fig. 3C). Importantly, the lowest E_sensor_ was obtained for MM_1_, which was also the closest MM to a sensor (Table 1 and Fig. 2).

### Effects of environmental factors

**Figure 4:**
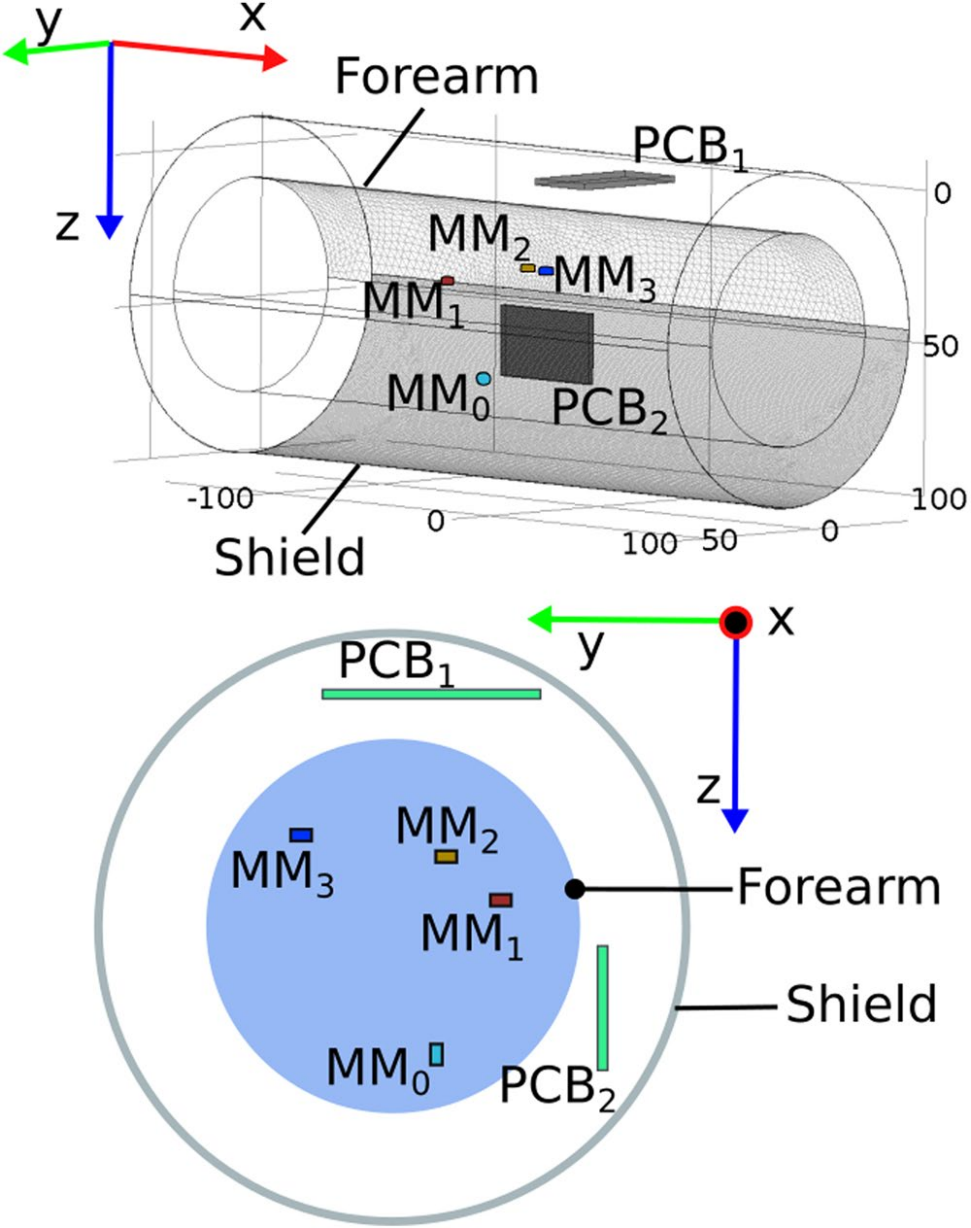
Finite Element magnetic model of the myokinetic control interface components. The human forearm was modelled as a solid water cylinder (length 250 mm, diameter 70 mm), with four implanted cylindrical MMs (MM_0_…MM_3_). The magnetic shield was modelled as a hollow cylinder (length 250 mm, diameter 110 mm, thickness 0.1 mm). The magneto-static disturbance (not shown) was modelled as a cubic magnet (L = 5 mm, Br = 14 kG) producing a 16 G field on the sensors on PCB1 (first simulation) or on PCB2 (second simulation) (i.e. 25 mm away from either PCB1 or PCB2).

**Figure 5:**
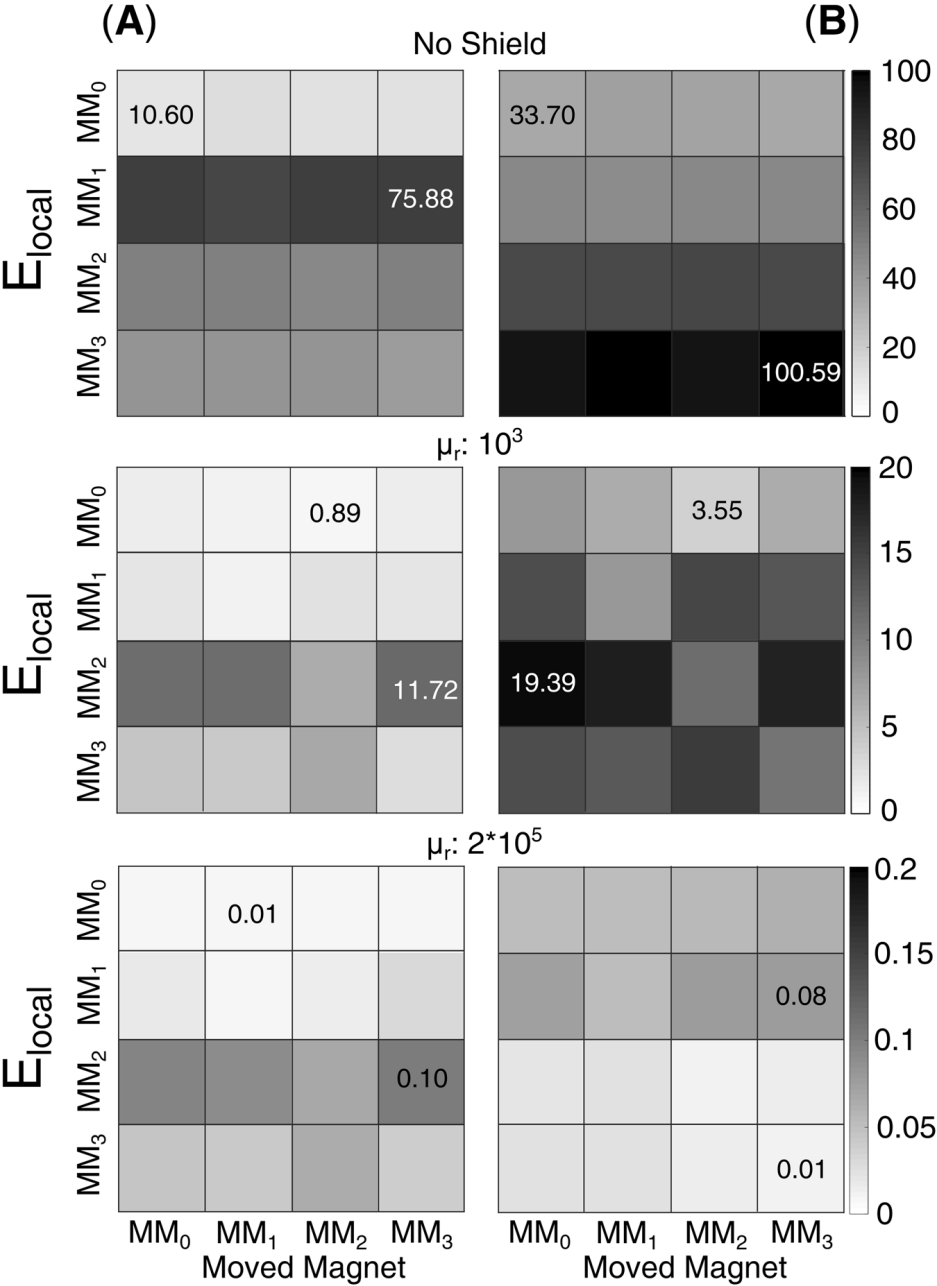
E_local_ due to 16 G magneto-static disturbance as a function of the relative permeability µ_r_ of the shield. Local disturbance on sensors on PCB_1_ (**A**) or on PCB_2_ (**B**). Each confusion matrix corresponds to a different µ_r_ of the shield. The figures in the confusion matrices indicate the minimum and maximum error (in mm) affecting that particular configuration.

The effect of a local magneto-static disturbance (E_local_ in Equation 3) and the possibility of attenuating it through a shield was evaluated through FE simulations (Fig. 4). The disturbance was modelled as a cubic permanent magnet (L = 5 mm, B_r_ = 10 kG) which produced a 16 G field on either the sensors on PCB1 or on PCB2. The shield was modelled as a hollow cylinder (0.1 mm thick, 25 cm long). The simulation was repeated changing the relative magnetic permeability ascribed to the shield (µ_r_ = 1, 10^3^, or 2∙10^5^, representing no-shield, steel and treated iron, respectively). The error induced by these disturbances, E_local_, was computed as the Euclidean distance between the positions estimated with and without the disturbances. As expected the local magneto-static disturbance severely affected the performance of the system, producing a maximum E_local_ (~100 mm) when the shield was not present (Fig. 5). Adding the shield helped improving the performance of the myokinetic interface; in particular, E_local_ decreased with increasing µ_r_, becoming as low as 80 µm in the case of µ_r_ = 2∙10^5^.

### Online performance/implementation

**Figure 6:**
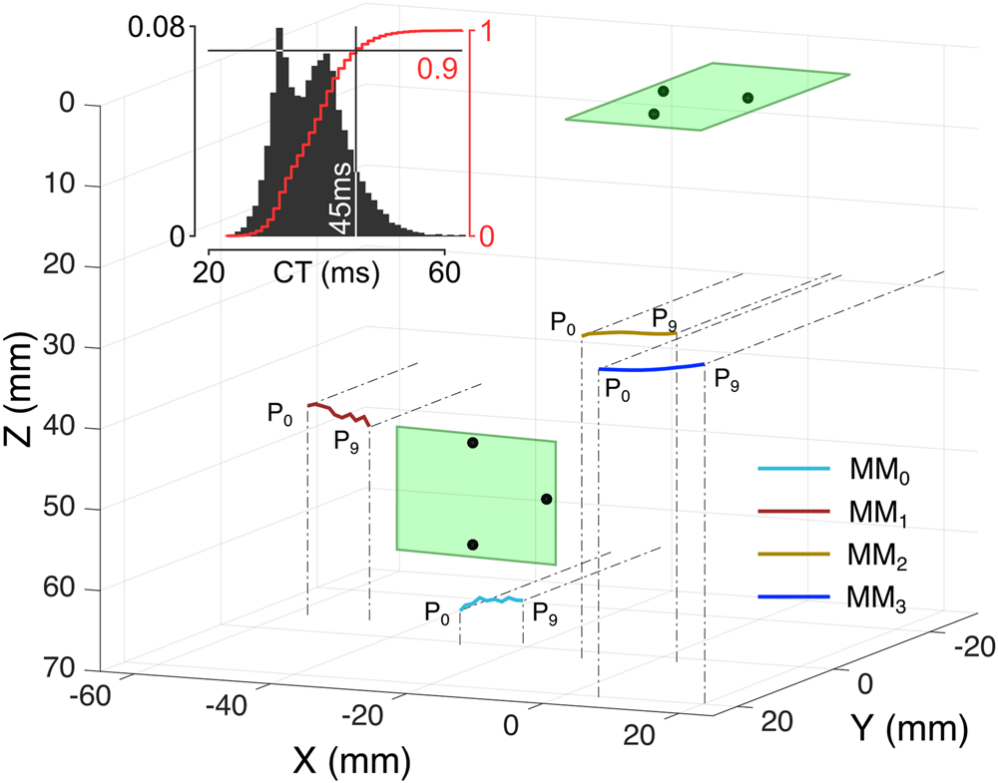
Online performance of the myokinetic control interface. Three-dimension representation of the trajectories of the four MMs reconstructed by the localizer. The system was able to track all MMs along their ROM. Magnets were never confused with each other. The relative position of the two PCBs with the magnetic field sensors are represented as green rectangles and black dots, respectively. The inset shows the distribution of the computation time (CT) i.e., the time required to compute the positions of four MMs resolving the inverse magnetic problem (Equation 2) using the Levenberg-Marquardt optimization method on the PC (Intel i7-6700 CPU running at 3.4 GHz, 16 GB of RAM, Windows 7). The red curve represents the cumulative distribution.

The computation time of the algorithms implemented in the prototype was evaluated by continuously moving all MMs and online estimating their position for 10 minutes, in order to collect a significant sample. The computation time proved larger than the sensor readout rate (i.e., 75 samples per second) and exhibited a bimodal distribution, with 90% of the samples being processed in less than 45 ms (Fig. 6). During this test, the myokinetic interface proved able to track the trajectories of the four MMs, without confusing the MMs with each other (Fig. 6). The prototype was thus used for an online demonstration of the capability to independently and proportionally control four DoFs of a robotic hand (supplementary Movie S1).

## Discussion

In this work we presented the idea of a *myokinetic* control interface, as a novel HMI for intuitive, direct control of multi-DoF prostheses. We developed a forearm mockup with the extrinsic muscles of the hand and experimentally assessed the performance of a six 3-axis sensor localizer, able to track the position of four MMs implanted in the mockup muscles. The promising results allowed to implement and demonstrate online, direct control, of four parallel DoFs of a robotic hand using the mockup and a data glove. If successfully translated to the clinical case, our outcomes would allow unprecedented, close to natural, controllability of powered prostheses.

The success of the myokinetic control interface relies on the ability of tracking the movements of the implanted MMs. In our experimental study, under ideal conditions (i.e. no external disturbances), E_model_ was found to be as large as 2.1 mm (Fig. 3A). While such an error might be unacceptable in the case of a system that has to exactly localize the magnets, in the context of the myokinetic controller it is only marginally important. Indeed, the functionality of the system does not depend on the ability to retrieve the exact displacement from the rest position but on the repeatability of the measurement. Remarkably, as long as the relationship between the actual and the estimated displacement is monotonic, the system works, and the precision is more important than the accuracy. Hence, E_sensor_, which affects the precision of the system, is a more appropriate metric to assess the performance. This was found to be very low in all conditions (<150 µm), yielding to highly repeatable measurements (Fig. 6). The high linearity found between the real and computed displacement (Fig. 3A) is important for direct control, as it means that the system can preserve the relationship between muscle contraction and the movement of the DoF in the prosthesis.

When a MM was moved, apparent displacements of the remaining MMs were also observed, when in fact they did not move (E_cross-talk_ up to 2.4 mm, Fig. 3). This effect could in principle produce undesired motion inter-dependencies among DoFs in the prosthesis, however the amount of cross-talk decreased with inter-MMs distance (Fig. 2). In the human forearm it would probably be possible to implant the MMs far enough to reduce the cross-talk; moreover, this issue could be limited using spherical MMs (instead of cylindrical ones), better approximated by the magnetic dipole model^22^.

Not surprisingly the simulations showed high sensitivity to external magnetic disturbances, akin to other systems^23^ (Fig. 5). However, such disturbances could be attenuated using a shield with appropriate relative magnetic permeability (µ_r_ = 2∙10^5^). Materials like treated iron exhibit such large permeability, and a shield with dimensions as the one modelled, would increase the weight of the prosthetic socket by ~65 g. In practice, very thin magnetic shielding materials with very high µ_r_ should be considered in order to ensure appropriate insulation from any magnetic disturbance.

The localizer was able to continuously compute positions of the four MMs at a 22 Hz rate, i.e., with delays of 45 ms, when running on a desktop PC. This value is well within the acceptable controller delay, and would go unnoticed to the users of a powered prosthesis^24^. The bimodal distribution found for the computational time indicates that the time to reverse Equation 2 depends on the solution, (i.e. spatial configuration of the MMs), akin to other numerical solvers^25^. Notably, the bottleneck of the system was the computational time and not the sensors readout stream. This suggests that using a faster CPU (and/or numerical algorithm or using completely different techniques, e.g. neural networks^26^) would increase the performance of the myokinetic controller, and leaves some space to the possibility of increasing the number of sensors in order to estimate additional information. In fact, with four magnets to be tracked by the localizer, a minimum of four 3-axis sensors could have been used, reducing the computation time even more. However, we chose to use more than four but not too many, in order to find a good trade-off between accuracy (due to data redundancy) and computation time (which increases with the number of sensors used). We invite further studies to investigate what is the optimal solution to this trade-off. While tracking the position of the MMs, we assumed constant and known the direction of their magnetic moments. Resolving the inverse magnetic problem also for the four orientations would require at least eight 3-axis sensors. We argue that the assumptions made in this study were a good first approximation of the clinical application, especially when considering MMs implanted at the distal end of the muscle, where the maximum translational displacement occurs^27^. However as small misalignments between the assumed orientation and the actual one lead to large errors^23^, in a future study we foresee to retrieve the orientation of the implanted MMs, using a larger number of sensors.

While the proposed myokinetic controller proved viable in a controlled experimental setup, several issues must be addressed before translating it into the clinical scenario. The first one is biocompatibility and biostability: magnets themselves are not. Hence it is necessary to make them biocompatible and bio-stable for long-term implantation, by coating or packaging them using materials like Parylene, titanium or gold. Neodymium magnets have been tested for medical uses such as magnetic braces and bone repair, but biocompatibility issues have prevented widespread application^28^. However, while uncoated samarium-cobalt (rare earth) magnets showed significant cytotoxicity, coated rare earth magnets showed good biocompatibility^29,30^. A second issue is the residual limb anatomical situation. After amputation, the contractile ability of the muscles may vary from subject to subject depending on (i) the level of amputation, and (ii) the initial amputation surgery^31^. For example, a trans-digit amputee, who maintains part of the muscle-tendon apparatus, is still able to bend part of the finger and therefore retains an excursion comparable to that of able-bodied subjects. The amount of excursion of the residual forearm muscles in the case of a more proximal amputation (e.g. transradial amputees) depends on whether the residual muscles are stabilized/stitched to the bone (i.e. myodesis) or to other muscles (myoplasty)^31^. The stiffer interface of the former solution allows for isometric contractions only, while in the latter case isotonic contractions can be performed as well. The mockup developed for this study replicated axial muscle deformations typically observed in healthy humans during isotonic contractions. Thus, whether this deformation can be observed in amputees remains to be assessed on a case by case basis. It should also be noted that the mockup did not emulate changes in muscles configuration due to forearm pronation-supination. Such changes could significantly affect the trajectories of the MM and in turn the control signals to the prosthesis, if not modelled. We argue that this effect may be rather limited, in practice. The prosthetic socket largely constrains and inhibits forearm pronation-supination movements, especially when the residual limb is long, and eventually, such movements could be tracked^23^.

The choice of replicating only axial deformations was dictated by the need to have a controllable and repeatable input signal (i.e. the muscle deformation), in order to finely characterize the myokinetic interface. Other systems, like pneumatic artificial muscles, could have been used, in order to better approximate volumetric muscles deformations. Nonetheless this solution was discarded because the deformation of pneumatic muscles is hardly controllable.

A clinical implementation requires to compensate for the effects of the relative compression/depression movements between the socket and the stump tissues (E_shift_ in Equation 3). Notably, such movements would cause an apparent similar displacement of all MMs simultaneously; this could be detected and cancelled out by dedicated algorithms. In addition, time-varying local magnetic interference generated by the motors in the prosthesis, could affect the system performance (increasing E_local_). Nonetheless as these interferences are likely to occur at frequency harmonics higher than those involved in human movements (<10 Hz^32^) they could be easily filtered out.

To conclude, to our knowledge this is the first study in which implanted magnetic markers are proposed for continuously tracking muscle contractions, in order to control multiple DoFs of a prosthetic hand. Our findings suggest indeed that the myokinetic control interface represents a concrete alternative to the use of neuromuscular electrical interfaces and open new paths towards the development of seamless HMIs.

## Materials and Methods

### Forearm mockup

In order to test the myokinetic interface, we built a physical mockup of the human forearm (Fig. 2). This aimed at reproducing the natural position and orientation of forearm muscles, in addition to their deformation due to contraction. Muscles were modelled using a wire (Fig. 2) attached on one side to a servo motor through a pulley and on the other side to a weight to maintain tension on the wire. Within this configuration, a rotation of the servo motor corresponded to a translation (contraction/elongation) of the wire (muscle) reproducing the axial deformation of such muscle during contraction. Muscles with multiple compartments were modelled using multiple wires, one for each compartment. Pulleys were used to guide the wires in a way they mimicked the position and orientation of the original model. The mockup replicated the movements of 17 degrees of freedom of the hand and wrist^33^: flexor and extensor carpi radialis, flexor digitorum superficialis (four compartments) and flexor digitorum profundus (four compartments), extensor digitorum comunis (four compartments), flexor and extensor pollicis longus and abductor pollicis longus. Based on the natural range of motion of the human joints^34^ and anthropometric data of healthy subjects^35^, we retrieved the range of natural contraction for each muscle and replicated it in the mockup. The mockup fitted into the size of a 75th percentile male forearm^35^ and was manufactured using diamagnetic materials in order to avoid magnetic interferences.

### Experimental setup

Four identical commercially available NdFeB cylindrical magnets (D = 4 mm, L = 2 mm, residual magnetic flux density B_r_ = 13.2–13.7 kG) were attached to four wires in the mockup, ideally associated to four muscles (Fig. 2). In particular, the MMs were attached to the abductor pollicis longus (MM_0_), flexor pollicis longus (MM_1_) and the compartments of the flexor digitorum superficialis inserting in the index (MM_2_) and middle fingers (MM_3_). The dimension of the magnets was optimized based on the geometry of the system^36^.

The localizer comprised two custom printed circuit boards (PCB), each equipped with three 3-axis magnetic field sensors and a host PC (Fig. 2). The PCBs could stream to the PC the readouts from the sensors at rate up to 75 samples per second, through a serial bus (RS-485). The PC (Windows 7, Intel i7–6700 CPU running at 3.4 GHz, 16 GB of RAM) used the sensors readouts to solve the magnetic inverse problem (Levenberg-Marquardt algorithm, implemented in a custom C# application) in order localize the four MMs. The geomagnetic field was cancelled out using differential measurements between each sensor and a remote sensor not subject to the magnetic field produced by the MMs.

In order to characterize the proposed interface, each MM was sequentially moved along its entire range of motion. Data was collected at ten equidistant locations (P_0_, …, P_9_, corresponding to relaxed muscle, …, maximum contraction) along the muscle trajectory (Fig. 2, lower panel). At each location, the mean (n = 80) displacement from P_0_ as retrieved by the localizer was calculated. E_model_ was then defined as the Euclidean distance between such displacement and the actual displacement imposed to the MM through the mockup (Fig. 2, lower panel). E_sensor_ was defined as the mean (n = 80) modulus of the standard deviation vector of the computed displacement. Finally, for all the other MMs (kept at P_0_), E_cross-talk_ was calculated as the mean (n = 10) displacement computed by the localizer.

The computation time of the implemented algorithms, which is important for the dynamic characterization of the myokinetic control interface, was measured during a 10 min session of continuous recording and online localization of the four MMs.

### Shielding of Magneto-static Interference

The capability of a magnetic shield in reducing quasi-static environmental magnetic interferences (contributing to E_local_ in Equation 3) was assessed through a Finite Element model (Comsol Multiphysics, COMSOL Inc.). The model (Fig. 4) comprised the MMs (having the same geometric characteristics and placed in the same spatial configuration of the physical ones used with the mockup), the forearm and the shield. The forearm was modelled as a solid cylinder (250 mm of length and 35 mm of radius) of magnetically homogeneous tissue having the magnetic permeability of water. The shield was modelled as a hollow cylinder (250 mm of length, 60 mm of radius and 1 mm of thickness) closed on the wrist side and concentric to the forearm. Within this configuration, we measured E_local_, computed as the Euclidean distance between the position retrieved by the localizer without the disturbance and the one when the disturbance was present. The measure was averaged among the same ten positions tested with the mockup (P_0_, …, P_9_). The disturbance was modelled as a cubic permanent magnet (L = 5 mm, B_r_ = 10 kG) placed 25 mm away from either PCB1 or PCB2, in two different simulations (Fig. 4). In particular, the distance was chosen in order to produce a 16 G modulus disturbance on each magnetic sensor (notably. this value was well beyond the range of the sensors if no shield was present). The simulation was repeated changing the relative magnetic permeability µ_r_ ascribed to the shield. In particular, µ_r_ was set to 1, 10^3^, or 2∙10^5^, for a total of 6 simulations (2 positions × 3 µ_r_). This range allowed to simulate the absence of the shield (µ_r_ = 1) and ferromagnetic materials like ferritic stainless steel (µ_r_ = 10^3^) and treated iron (e.g. 99.95% pure Fe annealed in H, µ_r_ = 2∙10^5^)^37^.

## Electronic supplementary material

Supplementary Information
